# Supplementary material for: Silver nanoparticle biosynthesis utilizing Ocimum kilimandscharicum leaf extract and assessment of its antibacterial activity against certain chosen bacteria
Source: PLoS One. 2024 May 29;19(5):e0295463. doi: 10.1371/journal.pone.0295463 (PMC11135695; doi:10.1371/journal.pone.0295463)
Supplement: S1 File — (ZIP) [file pone.0295463.s001.zip › Clarification.docx]

**For clarification:**
OK: *O. kilimandscharicum*;
AES: Aqueous Extract Silver;
MES: Methanolic Extract Silver.
