# Supplementary material for: Silver nanoparticle biosynthesis utilizing Ocimum kilimandscharicum leaf extract and assessment of its antibacterial activity against certain chosen bacteria
Source: PLoS One. 2024 May 29;19(5):e0295463. doi: 10.1371/journal.pone.0295463 (PMC11135695; doi:10.1371/journal.pone.0295463)
Supplement: Attachment — Submitted filename: Reviewer-comments to the author-Plos one-1.docx [file pone.0295463.s002.docx]

Reviewer’s Constructive Feedback to the Authors:

I have had the opportunity to review your study titled "**Silver nanoparticle biosynthesis utilizing *Ocimum kilimandscharicum* leaf extract and assessment of its antibacterial activity against certain chosen bacteria**." The abstract provides a clear overview of the study, which focuses on the use of Ocimum kilimandscharicum for the biological production of silver nanoparticles (AgNPs) and their potential antibacterial properties. There is no specific fault in the given text. However, here are some comments and suggestions:

1. There is inconsistency in the use of tenses. For example, the phrase "is becoming increasingly important" is in the present continuous tense, while the rest of the abstract is predominantly in the past tense.
2. A few typos in the document, such as aluminium instead of aluminum, space problem, comma and fullstop issues. Additionally, there are some other grammatical errors that may need attention.
3. The results and discussion sections are written together in this manuscript. I don't know if it falls within the rules of the journal or not. It is preferable to place this in different parts.
4. Picture quality should be increased, especially for Fig. 19a, b; For MIC and MBC they can include the picture of broth culture.

In this study the authors focuses on the synthesis of silver nanoparticles (AgNPs) using aqueous and methanolic extracts of Ocimum kilimandscharicum leaves. The UV-Vis analysis confirmed the presence of silver nanoparticles through the observation of their surface plasmon resonance. The biosynthesized AgNPs exhibited significant antibacterial activity against various pathogens. Considering the growing concerns regarding microbial resistance, AgNPs hold great potential as antimicrobial agents. Further research is recommended to investigate the in vitro and in vivo toxicity of these sustainable silver nanoparticles
