# Supplementary material for: Silver nanoparticle biosynthesis utilizing Ocimum kilimandscharicum leaf extract and assessment of its antibacterial activity against certain chosen bacteria
Source: PLoS One. 2024 May 29;19(5):e0295463. doi: 10.1371/journal.pone.0295463 (PMC11135695; doi:10.1371/journal.pone.0295463)
Supplement: Attachment — Submitted filename: Dear editor and author.docx [file pone.0295463.s003.docx]

**Dear editor and author**

Overall this is a good article that presents a novel synthesis strategy and good characterization techniques. However, I would like to make some suggestions.
I am attaching the manuscript with some comments I would like you to address.

1.There are lots of typo and grammatical mistakes in the manuscript making the manuscript less interesting to read.

2.IN FTIR studies, these functional groups indicated flavonoids, phenols, and ascorbic acid in Ocimum kilimandscharicum leaf extract-----FTIR can indicate only functional groups and not presence of compounds or metabolites.

3.XRD and EDX, and TEM added the manuscript.

4.The novelty of the research work in terms of prior art available has to be brought out.
