# Supplementary material for: Silver nanoparticle biosynthesis utilizing Ocimum kilimandscharicum leaf extract and assessment of its antibacterial activity against certain chosen bacteria
Source: PLoS One. 2024 May 29;19(5):e0295463. doi: 10.1371/journal.pone.0295463 (PMC11135695; doi:10.1371/journal.pone.0295463)
Supplement: Attachment — Submitted filename: Point per Point response to the reviewers.docx [file pone.0295463.s004.docx]

**Point per Point response to the reviewers.**

Reviewer(s)' Comments to Author:

Reviewer: 1

Comments:
I have had the opportunity to review your study titled "Silver nanoparticle biosynthesis utilizing Ocimum kilimandscharicum leaf extract and assessment of its antibacterial activity against certain chosen bacteria." The abstract provides a clear overview of the study, which focuses on the use of Ocimum kilimandscharicum for the biological production of silver nanoparticles (AgNPs) and their potential antibacterial properties. There is no specific fault in the given text.

**Answer: Thank you for the appreciation**

Questions:

There is inconsistency in the use of tenses. For example, the phrase "is becoming increasingly important" is in the present continuous tense, while the rest of the abstract is predominantly in the past tense.

**Answer: We thank the reviewer for the insightful comments and suggestions. We improved the the use of tenses in the manuscript. Thank you.**

A few typos in the document, such as aluminium instead of aluminum, space problem, comma and fullstop issues. Additionally, there are some other grammatical errors that may need attention.

**Answer: We thank the reviewer for the insightful comments and suggestions. We improved the quality of the document using your comment. We corrected the word aluminum, resolved the space problem and the grammatical errors. Thank you.**

The results and discussion sections are written together in this manuscript. I don't know if it falls within the rules of the journal or not. It is preferable to place this in different parts.

**Answer: We thank the reviewer for the insightful comments and suggestions. We checked PlosOne’s many articles, we have seen that both are accepted. Thank you.**

Picture quality should be increased, especially for Fig. 19a, b; For MIC and MBC they can include the picture of broth culture.

**We thank the reviewer for the insightful comments and suggestions. We increased the quality of pictures. Thank you very much.**

In this study the authors focuses on the synthesis of silver nanoparticles (AgNPs) using aqueous and methanolic extracts of Ocimum kilimandscharicum leaves. The UV-Vis analysis confirmed the presence of silver nanoparticles through the observation of their surface plasmon resonance. The biosynthesized AgNPs exhibited significant antibacterial activity against various pathogens. Considering the growing concerns regarding microbial resistance, AgNPs hold great potential as antimicrobial agents. Further research is recommended to investigate the in vitro and in vivo toxicity of these sustainable silver nanoparticles

**Answer: We thank the reviewer for the insightful comments and suggestions. Definitely. Considering the growing concerns regarding microbial resistance, AgNPs hold great potential as antimicrobial agents. We can reassure you the next step of our research is what you suggested. In vitro and in vivo toxicity of this sustainable silver nanoparticles. Thank you very much.**

Reviewer: 2

Questions:

Manuscript came out well.

**Answer: Thank you for the appreciation.**

In introduction, add some more points and literature review.

**Answer: We thank the reviewer for the insightful comments and suggestions. We added some points and literature review.**

In figure 2 Methanol crude extract graph is above the range, do it once again and add in the graph

**Answer: We thank the reviewer for the insightful comments and suggestions. We did it many times but we still got the same result. I think this due to the high concentration of the phytochemical’s compounds in the Methanol crude extract.**

You have added plain silver mean nano or silver nitrate; please express correctly.

**Answer: We thank the reviewer for the insightful comments and suggestions. It is the silver nitrate. But when we say the pain silver solution it means the silver nitrate. Thank you very much.**

Figure 3 only writes an OD in nm not an absorbance.

**Answer: We thank the reviewer for the insightful comments and suggestions. In literature UV/Vis Spectra is express like that. Absorbance (a.u.) and wavelength (nm). Thank you very much.**

You have performed the experiment with aqueous extract and methanol extract. need comparative study on all phytochemical analyses and which one is best to isolate bioactive molecules.

**Answer: We thank the reviewer for the insightful comments and suggestions. We did it in our previous study. We added it to the current manuscript. Thank you very much.**

In figure 6, why is there is peak in 360nm and what is the significance?

**Answer: We thank the reviewer for the insightful comments and suggestions. Certainly, the significance of the peak at 360 nm is undeniable. However, our research is centered on nanoparticles, which typically form within the wavelength range of 400 to 500 nm.**

Figure 8 and 9 what is a comparison interpretation? Add

**Answer: We thank the reviewer for the insightful comments and suggestions. One of the Reviewer suggested to delete these figures. Thank you very much.**

Only one application is done; some more applications like anti-cancer activity, environmental study, antioxidant activity.

**Answer: We thank the reviewer for the insightful comments and suggestions. Definitely. Considering the growing concerns regarding microbial resistance, AgNPs hold great potential as antimicrobial agents. We can reassure you the next step of our research is what you suggested. To add more application like anti-cancer activity, environmental study, antioxidant activity, in vitro and in vivo toxicity. Thank you very much.**

Reviewer: 3

Comments:

There are lots of typo and grammatical mistakes in the manuscript making the manuscript less interesting to read.

**Answer: We thank the reviewer for the insightful comments and suggestions. We improved the quality of the document using your comment. We corrected the grammatical errors. Thank you.**

In FTIR studies, these functional groups indicated flavonoids, phenols, and ascorbic acid in *Ocimum kilimandscharicum* leaf extract-----FTIR can indicate only functional groups and not presence of compounds or metabolites.

**Answer: We thank the reviewer for the insightful comments and suggestions.**

XRD and EDX, and TEM added the manuscript.

**Answer: We thank the reviewer for the insightful comments and suggestions. But we subbed them with the Dynamic Light Scattering using Malvern ZETASIZER NANO to determine the particle size, polydispersity index (PDI), and stability of biosynthesized AgNPs. And didn’t do it due to our limited budget.**

The novelty of the research work in terms of prior art available has to be brought out.

**Answer: In the literature, there are several studies that aimed to evaluate the medicinal and insecticidal properties of plants from *Ocimum* genus. However, the Kenyan cultivars have not yet been fully studied. We expect that the preliminary phytochemical screening of *Ocimum kilimandscharicum* and silver nanoparticle biosynthesis utilizing *Ocimum kilimandscharicum* leaf extract and assessment of its antibacterial activity could pave the way to the efficient use of this plant in human and animal health.**

Reviewer: 4

Comment:

The authors did interesting research work with a lot of results. thus, suggestion to combine or choose either figures or tables for some results. for example, FT-IR spectra are not needed when the table resumes all the results for it.

**Answer: We thank the reviewer for the insightful comments and suggestions. We removed the FT-IR spectra from the manuscript. Thank you very much.**
