# Supplementary material for: Silver nanoparticle biosynthesis utilizing Ocimum kilimandscharicum leaf extract and assessment of its antibacterial activity against certain chosen bacteria
Source: PLoS One. 2024 May 29;19(5):e0295463. doi: 10.1371/journal.pone.0295463 (PMC11135695; doi:10.1371/journal.pone.0295463)
Supplement: Attachment — Submitted filename: 1-Reviewer-comments to the author.docx [file pone.0295463.s005.docx]

Reviewer’s Constructive Feedback to the Authors:

I have had the opportunity to review your study titled "Genome-wide in silico characterization, validation, and cross-species transferability of microsatellite markers in Mallard and Muscovy ducks." Your research delves into the comparison of whole-genome and chromosome-wise microsatellite mining approaches in Muscovy and Mallard ducks, with a focus on assessing marker transferability between the two species. The assertion that you have developed a comprehensive whole-genome SSR panel for ducks, a pioneering feat, is a significant aspect of your work. Furthermore, your findings underscore the similarity between the outcomes of chromosome-wise mining and the holistic whole-genome approach.

However, I would like to bring to your attention a few areas that warrant attention for improvement:

1. Typographical Errors: There are several typographical errors present within your manuscript that require correction. Taking the time to thoroughly proofread and revise the text will undoubtedly enhance the clarity and professionalism of your work.
2. Referencing Tables: On page 6, in lines 149 to 155 and 199 to 211, it would be beneficial to include the corresponding table numbers that pertain to the discussed content. This will aid readers in cross-referencing your textual information with the relevant tables.
3. Contextual Significance: While your study primarily adopts a statistical analysis approach, I encourage you to delve into the broader significance of your findings beyond statistical comparisons. Demonstrating the potential implications of your results within the larger context of duck genetics or other related fields could amplify the relevance and impact of your work.
4. Expansion of the Study: To enhance the robustness of your research, I suggest considering the expansion of your work in either an in silico or in vivo setting. A more comprehensive comparison that encompasses these dimensions could offer deeper insights into the validity and applicability of your findings.

In conclusion, your study presents a commendable contribution to the field of duck genetics through its innovative exploration of microsatellite markers. Addressing the aforementioned points for refinement will undoubtedly bolster the overall quality and impact of your work. I look forward to witnessing the evolution of your study and its continued advancement.
